# Cost of Intravenous Analgesia for the Management of Acute Pain in the Emergency Department is Substantial in the United States

**DOI:** 10.36469/9793

**Published:** 2017-03-13

**Authors:** Pamela P. Palmer, Judith A. Walker, Asad E. Patanwala, Carin A. Hagberg, John A. House

**Affiliations:** 1 AcelRx, Redwood City, CA, USA; 2 QuintilesIMS, Alba Campus, Rosebank, Livingston, West Lothian, UK; 3 Department of Pharmacy Practice and Science College of Pharmacy, University of Arizona, Tucson, AZ, USA; 4 Department of Anesthesiology, UTHealth The University of Texas Health Science Center at Houston, McGovern Medical School, Houston, TX, USA; 5 Premier, Inc., Charlotte, NC, USA

**Keywords:** united states, costs, hydromorphone, fentanyl, morphine, nurse-controlled analgesia, emergency department, intravenous analgesia, opioids, acute pain

## Abstract

**Background:** Pain is a leading cause of admission to the emergency department (ED) and moderate-to-severe acute pain in medically supervised settings is often treated with intravenous (IV) opioids. With novel noninvasive analgesic products in development for this indication, it is important to assess the costs associated with IV administration of opioids.

**Materials and Methods:** A retrospective observational study of data derived from the Premier database was conducted. All ED encounters of adult patients treated with IV opioids during a 2-year time period, who were charged for at least one IV opioid administration in the ED were included. Hospital reported costs were used to estimate the costs to administer IV opioids.

**Results:** Over a 24 month-period, 7.3 million encounters, which included the administration of IV opioids took place in 614 US EDs. The mean cost per encounter of IV administration of an initial dose of the three most frequently prescribed opioids were: morphine $145, hydromorphone $146, and fentanyl $147. The main driver of the total costs is the cost of nursing time and equipment cost to set up and maintain an IV infusion ($140 ± 60). Adding a second dose of opioid, brings the average costs to $151-$154. If costs associated with the management of opioid-related adverse events and IV-related complications are also added, the total costs can amount to $269-$273. Of these 7.3 million encounters, 4.3 million (58%) did not lead to hospital admission of the patient and, therefore, the patient may have only required an IV catheter for opioid administration.

**Conclusions:** IV opioid use in the ED is indicated for moderate-to-severe pain but is associated with significant costs. In subjects who are discharged from the ED and may not have required an IV for reasons other than opioid administration, rapid-onset analgesics for moderate-to-severe pain that do not require IV administration could lead to direct cost reductions and improved care.

## BACKGROUND

Acute pain is a leading cause of admission to the emergency department (ED) in the United States,[1,2] and can be caused by a large spectrum of conditions including trauma, renal colic, burns, abdominal pain and myocardial infarction among others.

Treatment of pain is based on its intensity and should be individualized taking into account the patient's needs and response to initial analgesia.[3-10] Typically, pain is defined as mild, moderate or severe. Mild-tomoderate pain is usually managed with oral analgesics, while intravenous (IV) opioids, of which morphine, hydromorphone, and fentanyl are most commonly used in US EDs,[11,12] are generally reserved for moderateto- severe acute pain.[4,8-10,13,14] Despite the recognized efficacy of IV opioids, management of acute pain in the ED still remains a challenge;[15] pain is often under-recognized and under-treated, and in busy EDs, initiation of treatment may be delayed, leading to oligoanalgesia.[3] One of the potential causes of this apparent undertreatment of pain in the ED may be the resource use and time required to set up and administer IV opioids. In many cases IV opioid administration may be the only reason for IV cannulation. New noninvasive opioid delivery solutions which provide similar pain relief as the IV route, while requiring less resource utilization, are needed. These new treatment paradigms will need to consider costs as compared to the costs of current standard treatment.

Very few studies have assessed actual resource utilization and costs associated with IV administration of opioids in the United States[16,17] or indeed elsewhere (one study has been conducted in Hong Kong[18] and one had a European focus[19]).

The aim of this study was to determine the average total, fixed, and variable costs associated with IV opioid administration for the management of acute pain in the US ED setting. This could then serve as a basis for comparison of eventual new treatment modalities for pain in the ED.

## MATERIALS AND METHODS

A retrospective observational study of the Premier database was conducted to address the specific aims of the study.

The statistically de-identified Premier database (Premier, Inc.; Charlotte, NC) is a complete census of inpatients and hospital-based outpatients from geographically diverse hospitals (Table 1) and not a random sample. It contains data from more than 605 million patient encounters, approximately one in every five admissions in the nation. The database contains data from standard hospital discharge files, including a patient's demographic and disease state, and information on billed services, including medications, laboratory, diagnostics and therapeutic services in de-identified patient daily service records. In addition to the data elements available in most of the standard hospital discharge files, the Premier database also contains a day of service-stamped log of billed items, including procedures, medications, laboratory, and diagnostic and therapeutic services at the individual patient level. All procedures and diagnoses are captured for each patient, as well as all drugs and devices received. Drug utilization information is available by day of stay and includes quantity, charge per line item, strength used, and cost. As this is an analysis of de-identified claims data (in accordance with HIPAA (Health Insurance Portability and Accountability ACT of 1996)), this research was not subject to IRB (institutional review board) approval.

From the Premier database, encounters in the ED meeting the following inclusion criteria were selected: 1. ED encounter occurring between January 1, 2013 and December 31, 2014. 2. Age of patient ≥18 years, and 3. Charge for at least one IV opioid in the ED (morphine, hydromorphone, fentanyl, sufentanil, remifentanil, alfentanil, methadone, meperidine).

**Table 1.** Hospital Description Data

| | TOTAL N |
|---|---|
| # Unique Hospitals | 614 |
| Provider Region (n, %) | |
| Midwest | 130 (21.2%) |
| Northeast | 82 (13.4%) |
| South | 283 (46.1%) |
| West | 119 (19.4%) |
| Population Served (n, %) | |
| Rural | 160 (26.1%) |
| Urban | 454 (73.9%) |
| Teaching Hospital (n, %) | 169 (27.5%) |
| Hospital Size (# of beds) (n, %) | |
| < 100 | 148 (24.1%) |
| 100-199 | 128 (20.8%) |
| 200-299 | 120 (19.5%) |
| 300-399 | 85 (13.8%) |
| 400-499 | 56 (9.1%) |
| >500 | 77 (12.5%) |

The charge description file for hospitals identified by the patients in the total population was collated. From the charge description file the total, fixed, and variable costs of the following were identified: insertion of IV, morphine 5mg/mL in 1mL pre-filled disposable syringe, hydromorphone 1 mg/mL in 1 mL pre-filled disposable syringe, fentanyl 50 mcg/mL in a 2 mL vial, IV catheter, IV tubing, 250 mL saline bag, injectable 2% lidocaine, and an IV infusion pump (daily rental rate). These were based on what is optimally used to prepare for and administer an initial dose of opioid in a US ED department based on recent Institute of Safe Medical Practice (ISMP) guidelines for ED administration of IV opioids.[20] In this guideline, the ISMP advises that high-alert medications (which include IV opioids) be administered via fully-enabled programmable pumps. While there is variability of practice regarding IV administration of opioids from one institution to another, with some EDs routinely using lidocaine pre-treatment and IV infusion pumps, and others not, analyzing a comprehensive list of costs assesses the full burden of care, while still allowing the flexibility to disregard costs that do not apply to specific institutions or healthcare providers.

For costing, the top 3 prescribed IV opioids (morphine, hydromorphone and fentanyl) were the focus since the proportion of subjects receiving other opioids was very small (Table 2).

The cost for each item in the charge description file includes not only material acquisition costs (drug and non-drug costs), but also overhead costs, such as facility costs and charges, i.e., any costs incurred to deliver the drug to the patient. For morphine and hydromorphone, we assessed the total costs for prefilled syringes; for fentanyl, the cost of a 50 mcg/mL in a 2 mL single administration vial. We assumed equipotent dosing for each opioid and therefore analyzed 5 mg morphine, 1 mg hydromorphone and 100 mcg fentanyl (IV fentanyl's short duration of action requires 2 doses of 50 mcg to reach true dose equivalence).[21] An assumption was made that lidocaine local anesthesia was used for the initial IV catheter placement, which may not be the case in all instances, but the cost was relatively small and its use is relatively widespread. The cost of a 250 mL bag of saline was included for line flushing, priming the pump, and basal infusion to keep the line patent.

| | Total # Encounters | ED Visit | |
|---|---|---|---|
| | | Subject Admitted | Subject Not Admitted |
| | n = 7 327 299 (100%) | n = 3 055 611 (41.7%) | n = 4 271 688 (58.3%) |
| **IV Opioids Used** | | | |
| Morphine | 4 068 629 (55.5%) | 1 850 023 (60.5%) | 2 218 606 (51.9%) |
| Hydromorphone | 3 262 789 (44.5%) | 1 291 668 (42.3%) | 1 971 121 (46.1%) |
| Fentanyl | 1 856 870 (25.3%) | 1 446 810 (47.3%) | 410 060 (9.6%) |
| Meperidine | 258 800 (3.5%) | 139 421 (4.6%) | 119 379 (2.8%) |
| Sufentanil | 14 076 (0.2%) | 12 373 (0.4%) | 1703 (0.0%) |
| Remifentanil | 10 534 (0.1%) | 9835 (0.3%) | 699 (0.0%) |
| Alfentanil | 3047 (0.0%) | 2326 (0.1%) | 721 (0.0%) |
| Methadone | 1615 (0.0%) | 1479 (0.0%) | 136 (0.0%) |

ED: Emergency Department, IV: intravenous

Descriptive statistics were used to gain a better understanding of patients presenting to the ED and receiving IV opioids. Data measured on a continuous scale were expressed as mean ± standard deviation and categorical data were expressed as counts and percentages. Across hospitals, the individual costs are presented as mean ± standard deviation, and median with interquartile range. Costs were inflation-adjusted to 2016 USD using the US Department of Labor Consumer Price Index-All Urban Consumers data for Medical Care for the first half of 2016.[22] To remove extreme outliers, costs were Winsorized by trimming to the 1$^{st}$ and 99$^{th}$ percentile.[23] All analyses were performed using SAS version 9.4 (SAS Institute, Cary, NC). Total costs were summarized by initially summing all IV set-up and administration costs within a hospital, excluding the cost of the IV opioid, and averaging across the hospitals (total cost without drug, i.e. fixed costs before a single dose of drug is administered, which includes non-drug materials, nursing time, administrative costs, etc). Then the cost of each of the three most commonly administered IV opioids (morphine, hydromorphone, or fentanyl) was added to assess the total cost of administering an initial IV opioid dose.

The primary analysis did not include the cost of management of key adverse events (AEs) and IV complications associated with IV administration of opioids; however these have been considered in previous publications[19] and are estimated from the literature for the US population to provide a broader assessment of true costs.

## RESULTS

### Demographics

Table 3 indicates that a total of 7 327 299 encounters occurred during the 24-month time frame of which 3 055 611 (41.7%) led to inpatient admission.

| | ED Encounters receiving IV Opioids | | |
|---|---|---|---|
| | Total | ED Visit | |
| | | Subject Admitted | Subject Not Admitted |
| | n = 7 327 299 (100%) | n = 3 055 611 (41.7%) | n = 4 271 688 (58.3%) |
| Age | 51.2 ± 18.9 | 59.2 ± 18.8 | 45.5 ± 16.9 |
| **Age** | | | |
| ≤40 | 2 373 634 (32.4%) | 555 197 (18.2%) | 1 818 437 (42.6%) |
| 41-49 | 1 159 217 (15.8%) | 362 496 (11.9%) | 796 721 (18.7%) |
| 50-59 | 1 364 037 (18.6%) | 580 016 (19.0%) | 784 021 (18.4%) |
| 60-69 | 1 030 544 (14.1%) | 572 869 (18.7%) | 457 675 (10.7%) |
| 70-79 | 738 193 (10.1%) | 486 467 (15.9%) | 251 726 (5.9%) |
| 80 + | 661 674 (9.0%) | 498 566 (16.3%) | 163 108 (3.8%) |
| **Sex** | | | |
| Female | 4 319 350 (58.9%) | 1 646 230 (53.9%) | 2 673 120 (62.6%) |
| Male | 3 007 845 (41.0%) | 1 409 356 (46.1%) | 1 598 489 (37.4%) |
| Unknown | 104 (0.0%) | 25 (0.0%) | 79 (0.0%) |
| **Race** | | | |
| Caucasian | 4 929 262 (67.3%) | 2 064 879 (67.6%) | 2 864 383 (67.1%) |
| African American | 1 212 943 (16.6%) | 485 671 (15.9%) | 727 272 (17.0%) |
| Other | 1 181 680 (16.1%) | 503 847 (16.5%) | 677 833 (15.9%) |
| Unknown | 3414 (0.0%) | 1214 (0.0%) | 2200 (0.1%) |
| **Ethnicity** | | | |
| Hispanic or Latino | 716 806 (9.8%) | 262 121 (8.6%) | 454 685 (10.6%) |
| Not Hispanic or Latino | 5443 978 (74.3%) | 2 257 555 (73.9%) | 3 186 423 (74.6%) |
| Unknown | 1 166 515 (15.9%) | 535 935 (17.5%) | 630 580 (14.8%) |
| **Payor** | | | |
| Commercial | 2 253 848 (30.8%) | 773 714 (25.3%) | 1 480 134 (34.6%) |
| Medicaid | 1 361 597 (18.6%) | 461 908 (15.1%) | 899 689 (21.1%) |
| Medicare | 2 524 520 (34.5%) | 1 516 382 (49.6%) | 1 008 138 (23.6%) |
| Any Other Payor | 1 187 334 (16.2%) | 303 607 (9.9%) | 883 727 (20.7%) |
| **Provider Region** | | | |
| Midwest | 1 306 891 (17.8%) | 531 034 (17.4%) | 775 857 (18.2%) |
| Northeast | 878 626 (12.0%) | 516 365 (16.9%) | 362 261 (8.5%) |
| South | 3 874 641 (52.9%) | 1 532 268 (50.1%) | 2 342 373 (54.8%) |
| West | 1 267 141 (17.3%) | 475 944 (15.6%) | 791 197 (18.5%) |
| **Population Served** | | | |
| Rural | 1 067 561 (14.6%) | 351 359 (11.5%) | 716 202 (16.8%) |
| Urban | 6 259 738 (85.4%) | 2 704 252 (88.5%) | 3 555 486 (83.2%) |
| Teaching Hospital | 2 531 942 (34.6%) | 1 238 233 (40.5%) | 1 293 709 (30.3%) |
| **Hospital Size (# beds)** | | | |
| < 100 | 594 175 (8.1%) | 141 | 466 (4.6%) |
| 100 - 199 | 1 152 957 (15.7%) | 421 097 (13.8%) | 731 860 (17.1%) |
| 200 - 299 | 1 370 148 (18.7%) | 559 611 (18.3%) | 810 537 (19.0%) |
| 300 - 399 | 1 350 690 (18.4%) | 547 403 (17.9%) | 803 287 (18.8%) |
| 400 - 499 | 1 028 371 (14.0%) | 447 343 (14.6%) | 581 028 (13.6%) |
| ≥ 500 | 1 830 958 (25.0%) | 938 691 (30.7%) | 892 267 (20.9%) |

ED: Emergency Department; IV: intravenous

The mean age (+/- SD) of treated subjects was 51.2 ± 18.9 years. Not surprisingly, the age of admitted patients was higher (59.2 ± 18.8) than that of the cohort which was eventually discharged (45.5 ± 16.9) ($p<0.001$, for all comparisons between hospital admissions and ED-discharged subjects).

Slightly more female subjects presented to the ED, but relatively more males were admitted; the distribution of race across the entire cohort was reflective of the US population.

Relatively more Medicare patients were admitted in reference to the proportion of Medicare patients receiving IV opioids, which may be a reflection of their older age (Table 3).

Hospitals from the South contributed more data relative to other regions, though a geographic cross-section of subjects and hospitals throughout the US is represented. A cross section of hospital size, teaching status and urban vs rural location, consistent with US landscape are in the database (Table 1).

### IV Opioids Used in the Emergency Department

Morphine, hydromorphone and fentanyl were the three most commonly administered IV opioids (Table 2). Although morphine was the most commonly administered opioid overall, in the group that eventually was admitted, there was a substantial relative increase in use of fentanyl.

### IV Opioid Administration Costs

Costs were provided for each parameter in Table 4 and used to calculate total costs. For the primary calculations, costs of a single initial administration were summed, with and without inclusion of drug costs (Table 5).

This analysis reveals that the cost (mean ± SD) of administering an initial IV opioid dose in the ED setting was \$140 ± 60, before any actual drug costs are included (i.e., this fixed cost includes equipment cost, nursing time, overhead, etc). When the cost of morphine was included, the cost was \$145 ± 72; when hydromorphone was added, \$144 ± 66; and when fentanyl is used, \$147 ± 66. It is clear from this, that the costs are driven by nursing time and equipment costs, rather than by the costs of the drug itself (Figure 1).

**Table 4.** Individual Costs (2016 USD)

| | Total N |
|---|---|
| Number of Unique Emergency Departments | 614 |
| **Cost** | |
| **IV Start and Infusion** | |
| Mean (Std Dev) | 62.31 ± 4.99 |
| Median (IQR) | 62.40 (60.26, 65.46) |
| Minimum | 51.07 |
| Maximum | 69.23 |
| **Morphine 5 mg/ml in 1 mL pre-filled disposable syringe** | |
| Mean (Std Dev) | 5.61 ± 5.61 |
| Median (IQR) | 4.07 (2.11, 7.27) |
| Minimum | 1.40 |
| Maximum | 46.48 |
| **Hydromorphone 1 mg/ml in 1 mL pre-filled disposable syringe** | |
| Mean (Std Dev) | 6.58 ± 5.24 |
| Median (IQR) | 4.55 (2.79, 9.01) |
| Minimum | 1.40 |
| Maximum | 25.00 |
| **Fentanyl 50 mcg/mL, 2 mL vial** | |
| Mean (Std Dev) | 7.01 ± 4.75 |
| Median (IQR) | 5.55 (3.56, 10.00) |
| Minimum | 1.50 |
| Maximum | 25.99 |
| **IV Catheter** | |
| Mean (Std Dev) | 3.68 ± 3.69 |
| Median (IQR) | 2.59 (2.17, 3.36) |
| Minimum | 0.47 |
| Maximum | 22.50 |
| **250ml Saline Bag** | |
| Mean (Std Dev) | 14.47 ± 8.25 |
| Median (IQR) | 13.43 (9.25, 18.73) |
| Minimum | 1.35 |
| Maximum | 47.11 |
| **Pump Tubing** | |
| Mean (Std Dev) | 15.31 ± 6.76 |
| Median (IQR) | 18.19 (7.90, 20.80) |
| Minimum | 4.74 |
| Maximum | 25.06 |
| **IV Infusion Pump** | |
| Mean (Std Dev) | 36.88 ± 28.50 |
| Median (IQR) | 26.34 (22.69, 63.66) |
| Minimum | 1.91 |
| Maximum | 109.94 |

**Table 4.** Individual Costs (2016 USD) (continued)

| | Total<br>N |
|---|---|
| **Cost** | |
| **2% Lidocaine** | |
| Mean (Std Dev) | 4.90 ± 3.43 |
| Median (IQR) | 4.10 (2.56, 5.92) |
| Minimum | 0.68 |
| Maximum | 23.49 |
| **Total Cost (excluding IV morphine/hydromorphone/fentanyl)** | |
| Mean (Std Dev) | 137.60 ± 59.25 |
| Mean (95% CI) | 137.60 (132.91-142.29) |
| Median (IQR) | 111.20 (104.80, 177.90) |
| Minimum | 60.22 |
| Maximum | 297.33 |
| **Total Cost (with morphine)** | |
| Mean (Std Dev) | 143.20 ± 70.53 |
| Mean (95% CI) | 143.20 (137.62-148.78) |
| Median (IQR) | 115.30 (106.90, 185.20) |
| Minimum | 61.62 |
| Maximum | 343.81 |
| **Total Cost (with hydromorphone)** | |
| Mean (Std Dev) | 144.20 ± 65.15 |
| Mean (95% CI) | 144.20 (139.05-149.35) |
| Median (IQR) | 115.70 (107.60, 186.90) |
| Minimum | 61.62 |
| Maximum | 322.33 |
| **Total Cost (with fentanyl)** | |
| Mean (Std Dev) | 144.60 ± 65.38 |
| Mean (95% CI) | 144.60 (139.43-149.77) |
| Median (IQR) | 116.80 (108.40, 187.90) |
| Minimum | 61.72 |
| Maximum | 323.32 |

USD: United States dollars; IV: intravenous

Due to IV set-up equipment and nurse resources, costs are mainly incurred with the first dose and therefore each subsequent dose of opioid is relatively less costly, however, even when a second dose is added, the average per dose costs are still considerable. Based on several ED studies[12,24-26] in the United States and in line with recommendations from clinical practice guidelines,[4,8-10,27] patients presenting to the ED with acute pain typically spend 90 minutes in the ED[28] and receive 2 standard doses of opioid (the equivalent of 0.1 mg/kg, or approximately 10 mg of IV morphine).

The cost is $151 for 2 doses of morphine, $153 for hydromorphone and $154 for fentanyl, with an average per dose cost therefore of approximately $75. Similarly, the per dose cost would decrease further with subsequent doses.

**Table 5.** Total Costs (2016 USD)

| | Total for Single Administration N |
|---|---|
| **# Unique Emergency Departments** | 614 |
| **Cost** | |
| **Total Cost (excluding IV morphine/hydromorphone/fentanyl)** | |
| Mean (Std Dev) | 139.66 ± 60.14 |
| Median (IQR) | 112.86 (106.37, 180.56) |
| **Total Cost (with morphine)** | |
| Mean (Std Dev) | 145.34 ± 71.58 |
| Median (IQR) | 117.02 (108.50, 187.97) |
| **Total Cost (with hydromorphone)** | |
| Mean (Std Dev) | 146.36 ± 66.12 |
| Median (IQR) | 117.43 (109.21, 189.70) |
| **Total Cost (with fentanyl)** | |
| Mean (Std Dev) | 146.76 ± 66.36 |
| Median (IQR) | 118.55 (110.02, 190.71) |

USD: United States dollars; IV: intravenous

| Costing input | Rates for morphine | Rates for hydromorphone | Rates for fentanyl | Sources |
|---|---|---|---|---|
| **Management of adverse events (rate)** | | | | |
| Nausea | 12.1% | 21.1% | 19.0% | Beaudoin 2014,[30] Behzadnia 2012,[31] Bijur 2006,[32] Birnbaum 2007,[33] Birnbaum 2012,[24] Chang 2006,[34] Chang 2009,[35] Evans 2005,[36] Fleichsman 2010,[25] Galinski 2005,[37] Silverman 2004,[38] Soleimanpour 2012,[39] Vergnion 2001,[40] Zare 2014[41] |
| Vomiting | 5.8% | 9.5% | 10.7% | Azizkhani 2013,[42] Bijur 2006,[32] Birnbaum 2007,[33] Birnbaum 2012,[24] Chang 2006,[34] Chang 2009,[35] Evans 2005,[36] Galinski 2005,[37] Silverman 2004,[38] |
| **Soleimanpour 2012,39 Vergnion 200140** | | | | |
| Hypotension | 2.1% | 1.6% | 1.1% | Azizkhani 2013,[42] Bektas 2009,[43] Chang 2009,[34] Chang 2006,[35] Fleichsman 2010,[25] Fry 2002,[44] Serinken 2012,[45] Soleimanpour 2012[39] |
| Respiratory depression | 1.1% | 1.1% | 1.1% | Bektas 2009,[43] Chang 2009,[35] Evans 2005,[36] Fleichsman 2010,[25] Fry 2002,[44] Lvovschi 2008,[46] O'Connor 2000,[47] Serinken 2012,[45] Zare 2014[41] |
| **IV complications treatment* (rate)** | | | | |
| Phlebitis | 2.6% risk | 2.6% risk | 2.6% risk | Cicolini 2014[48] |
| Extravasation | 0.4% risk | 0.4% risk | 0.4% risk | Rupp 2015,[49] Adami 2005,[50] Watanabe 2008,[51] Hardie 2014[52] |
| Harmful IV prescribing errors | 1.2% risk | 1.2% risk | 1.2% risk | Davies 2011[53] |

AE: adverse event; IV: intravenous. AE and complication rate for each opioid as well as the sources of these data are provided in the table above. *It does not include complications specifically related to use of infusion pump.

These primary calculations are not all-inclusive; in particular, they do not include costs of management of AEs or IV complications. We have estimated these, based on literature review which has been, in part, previously reported.[19] As displayed in Table 6, Table 7, and Table 8, costs for the management of the most common AEs and IV complications were estimated at $118 for morphine, $119 for hydromorphone, and $118 for fentanyl.

Therefore, adding the costs of management of AEs and IV complications leads to considerable costs, ranging from $269 to $273.

| | **Costs per unit in 2016 USD** |
|---|---|
| **Adverse Events** | |
| Nausea* | $11.94 |
| Vomiting* | $21.09 |
| Hypotension* | $5.88 |
| Respiratory depression* | $904.51 |
| **IV complications** | |
| Phlebitis* | $4.70 |
| Extravasation* | $4.70 |
| IV prescription errors* | $8661.63 |

AE: adverse event; IV: intravenous; ED: emergency department; USD: United States dollars
Costs are given as 2016 USD adjusted costs. *Costs are given per episode. Methodology used is as per DiDonato et al 2016,[19] except for the treatment of nausea and vomiting in which ondansetron was substituted for metoclopramide to reflect the more common use of this agent in the United States.

| **Costing Input** | **Cost (2016 USD) IV opioid (weighted)** |
|---|---|
| **Management of adverse events** | |
| Nausea | 2.49 |
| Vomiting | 2.15 |
| Hypotension | 0.13 |
| Respiratory depression | 12.43 |
| **IV complications treatment** | |
| Phlebitis | 0.12 |
| Extravasation | 0.02 |
| Harmful IV prescribing errors | 104.99 |
| **Aggregated costs** | |
| AEs and IV complications | 121.90 |

AE: adverse event; IV: intravenous; ED: emergency department
Costs were derived from the product of the rates for each drug (Table 6) and the cost of management of each event (Table 7).

## DISCUSSION

The current analysis highlights the substantial cost of IV opioid administration for moderate-to-severe acute pain in the ED setting in the United States. The cost of administering an initial IV opioid dose for acute pain in the ED ranged between $145 and $147, when considering the costs of the analgesic, materials and workforce. These costs rise to between $151 and $154 when considering the more usual situation in which a second dose is administered, and to $269-$273 when costs for the management of adverse events and IV complications are included. In some cases in which IV opioids were administered, the IV cannulation may be done for the sole purpose of administering IV opioids. In other cases, such as multiple trauma, IV cannulation would be required in any case to administer fluids and other agents.

Although acute pain is a leading cause of referral to the ED, very few studies have assessed the economic burden of its management in this setting. Given that the majority of the cost of administering IV opioids is in the initial set-up of the IV line, newer noninvasive analgesics may prove to be substantially cost-saving for patients with acute pain in the ED but otherwise not requiring an IV line for medical management.[29]

A previous study was conducted to evaluate comparative costs of analgesia in the ED, including IV morphine.[16] Costs associated with IV morphine amounted to US$33 (2008 values; $37 when inflated to 2016 values). Costs were estimated based on published literature and primary interviews with ED staff, rather than primary database analysis of actual costs, as in the current study. The input parameters in their model included costs of medication, IV bolus materials, workforce time and management of two adverse events (nausea and vomiting). Pump infusion costs were not included. The costs associated with nursing time required for IV morphine administration differ significantly between that of the previous work ($15) and the current study ($62). These differences may relate to the source of the data.

The authors of the current paper have evaluated costs of treating moderate-to-severe pain in the ED in 5 European countries: France, Germany, Italy, Spain, and the United Kingdom.[19] Since there is no EU-based database similar to Premier's database from which to analyze data, a micro-costing approach was taken to estimate costs. This EU study revealed total estimated costs of €121-€132 ($138-$150) to manage an episode of acute pain. The main driver of the total costs in these EU countries was the cost of management of IV-related complications (phlebitis, extravasation and IV prescription errors), which accounted for 73% of the total costs.

Similar to other models, the current study has a number of limitations. Firstly, an assumption was made that any encounter in the ED, which led to administration of an IV opioid was for a pain complaint. While this is probably true in most cases, there will undoubtedly be a small percentage of subjects who presented with non-painful conditions, such as pulmonary edema due to congestive heart failure, in which IV morphine may be used.

Our analysis was simplified by assuming that all patients reach pain relief with an overall dose of opioid equivalent to 10 mg IV morphine. In clinical practice patients often receive supportive treatment with oral analgesics and in a considerable percentage of patients, doses higher than 10 mg of morphine are needed.[12] This assumption could therefore lead to an underestimation of total costs and an overestimation of cost per dose. In addition, wastage of drug was not included in the calculations.

Additional assumptions were made about drug usage, including which dosage forms were used, the use of lidocaine as a local anesthetic prior to catheter placement and the use of a programmable infusion pump based on recent ISMP guidelines for IV opioid administration as described earlier.[20] Incorrect assumptions may lead to slight changes in real costs, but these changes would be expected to be small.

An additional limitation of our analysis is the lack of formal assessment of AE management costs from the Premier data and the reliance on AEs and IV complication estimates drawn from a literature review. Including those costs provides a better reflection of the true overall cost of IV opioid administration. The approach to determine the costs for AEs and IV complications was conservative, only including the costs for a reduced number of typical opioid adverse events (nausea, vomiting, hypotension and respiratory depression) and IV-related complications (phlebitis, extravasation and harmful IV prescribing errors). Costs were not included for pump malfunction. The model does not reflect the contribution of other adverse events, such as sedation or pruritus or IV-related complications such as needle-stick injuries. Their inclusion would have increased the costs of IV-administered opioids further.

## CONCLUSIONS

In summary, the resources required for IV opioid administration in EDs are substantial and the associated costs previously not well understood. This analysis is based on a large database of actual cost data which lends further credence to the main results of considerable costs of IV opioid administration.

Novel non-invasive analgesics that can be administered using a less burdensome route could reduce significantly the costs of analgesia for moderate-to-severe acute pain in the ED setting. It might be relevant to repeat this analysis, once such newer treatments are available in the US. This current analysis provides a baseline assessment of current treatment costs of IV opioids in US emergency departments to which a cost assessment of new treatments could be compared.

## COMPETING INTERESTS

The research described in this article, the drafting of this manuscript and its publication were funded by AcelRx. The work of QuintilesIMS Consulting and Premier was funded by AcelRx. JW, AP, CH and JH are consultants to AcelRx. PP is an employee of AcelRx. All authors have participated in the design, data analysis and drafting of this article.

## ACKNOWLEDGEMENTS

The authors wish to thank Pim Kooreman and Montserrat Casamayor for assistance in preparing the manuscript.

## Figures and Tables

**Table 1.** Hospital Description Data

**Table 2.** Intravenous Opioids Administered in the ED

**Table 3.** Demographics of Patients receiving IV Opioids in the ED and Hospitals providing ED Care

**Figure 1.** Contribution of the Components to the Overall Costs, assuming Administration of 2 IV Opioid Doses

**Table 4.** Individual Costs (2016 USD)

**Table 5.** Total Costs (2016 USD)

**Table 6.** Summary of Non-cost Input Parameters for AEs and IV Complications

**Table 7.** Summary of Costs to Manage each AE and IV Complication in US Emergency Departments

**Table 8.** Estimated Cost for Management of AEs and IV Complications associated with IV Opioid Use in the ED (weighted for relative frequency for morphine, hydromorphone, fentanyl)

